# Feasibility and Acceptability of a Psychological Intervention for Internalized Health-Related Stigma Among Adults With Chronic Health Conditions: Preliminary Investigation

**DOI:** 10.2196/69548

**Published:** 2025-07-29

**Authors:** Rebecca L Pearl, Danielle Saunders, Laurie C Groshon, Yulin Li, Abigail Shonrock, Rebecca M Puhl, Kimberly A Driscoll, Preeti Manavalan, Joel M Gelfand, Thomas A Wadden, Sarah C Westen, Marjorie Montanez-Wiscovich, Xiang-Yang Lou

**Affiliations:** 1 Department of Clinical and Health Psychology, College of Public Health and Health Professions, University of Florida, P.O. Box 100165, Gainesville, FL, 32610-0165, United States, 1 3522945405; 2 Department of Biostatistics, College of Medicine and College of Public Health and Health Professions, University of Florida, Gainesville, FL, United States; 3 Department of Human Development & Family Sciences, University of Connecticut, Hartford, CT, United States; 4 Department of Medicine, College of Medicine, University of Florida, Gainesville, FL, United States; 5 Department of Dermatology and Department of Biostatistics, Epidemiology and Informatics, Center for Clinical Sciences in Dermatology, University of Pennsylvania Perelman School of Medicine, Philadelphia, PA, United States; 6 Center for Weight and Eating Disorders, Department of Psychiatry, University of Pennsylvania Perelman School of Medicine, Philadelphia, PA, United States; 7 Department of Dermatology, College of Medicine, University of Florida, Gainesville, FL, United States

**Keywords:** cancer, chronic disease, diabetes, HIV, obesity, pain, skin disease, stigma

## Abstract

**Background:**

Health-related stigma is widely acknowledged as a threat to public health and a barrier to managing chronic health conditions. Internalized stigma is a particularly strong predictor of poor health outcomes across health conditions, yet few evidence-based interventions are available. Peer support and counseling have been investigated as interventions for reducing internalized stigma. Typically, these interventions are developed and tested in disease-specific research, focusing on one health condition in isolation from others. This approach may limit knowledge and dissemination of support for health-related stigma across health conditions. Recent work has highlighted the need for research that breaks down traditional silos by using cross-cutting approaches to understand and reduce stigma.

**Objective:**

This study aimed to determine the feasibility and acceptability of a new group-based psychological intervention designed to reduce internalized health-related stigma among adults with different stigmatized chronic physical health conditions.

**Methods:**

A group intervention that was initially designed to address internalized weight stigma was adapted to be generalizable to other forms of internalized health-related stigma. This was done with input from Advisory Board members living with different stigmatized chronic health conditions and health professionals who specialized in these conditions. Adults with obesity, diabetes, HIV, skin diseases, chronic pain, or cancers were recruited to attend 12 weekly online group meetings. The average session attendance rate was computed with and without makeup sessions. A treatment acceptability questionnaire was completed at week 12. Primarily for feasibility testing, participants completed pre- and post-treatment questionnaires that assessed internalized health-related stigma and other relevant aspects of mental health and health-related quality of life. At baseline, participants were also asked to report reasons for perceived discrimination. Data collection occurred from December 2023 through April 2024.

**Results:**

In total, 10 adults were recruited within approximately 6 weeks, of whom 8 attended at least 1 treatment session and completed post-treatment questionnaires, with a 80% retention. The average session attendance rate was 95.8% with makeup sessions and 83.3% for those without makeup. Treatment acceptability ratings were high, with an overall acceptability rating of approximately 6.5 (SD 0.5) out of 7. Medium to large effect sizes were observed for changes in internalized stigma and some aspects of mental health. Almost all (n=7, 87.5%) of participants reported experiencing discrimination due to their health conditions, which accompanied a wide range of other reasons for perceived discrimination.

**Conclusions:**

Results showed high feasibility and acceptability of a transdiagnostic, online group psychological intervention for internalized health-related stigma delivered to adults with different types of stigmatized chronic physical health conditions. Given the small sample size and limited generalizability, testing in a large efficacy trial is needed to determine intervention benefits.

## Introduction

Health-related stigma involves negative judgment, blame, social rejection, and discrimination due to health conditions [1]. Stigma has been well documented across many types of chronic medical conditions that vary in key characteristics, such as their visibility (vs concealability), perceived risk of contagion, and perceived controllability [2,3]. For example, adults with obesity, type 1 or type 2 diabetes, skin diseases, HIV, chronic pain, and cancers face pervasive stigma for their health conditions [1,4-6].

In addition to experiencing stigma from other people, negative attitudes can be internalized by adults with these health conditions, contributing to self-blame and self-devaluation (ie, self-stigma) [7,8]. For example, individuals with obesity who internalize weight stigma often apply negative stereotypes to themselves (eg, believing that they are lazy or lack willpower) and blame themselves for their weight, leading them to view themselves as a failure [9]. The internalization of health-related stigma is associated with poor mental and physical health outcomes across different health conditions, including increased loneliness, depression, and anxiety, and reduced quality of life [1]. Internalized health-related stigma is also linked to reduced engagement in self-management behaviors for chronic health conditions—such as less healthy eating and physical activity, impaired medication adherence, and avoidance of medical appointments—which can further contribute to worse health outcomes [9-13]. Internalized stigma due to health conditions can interact with stigma related to other aspects of a person’s identity, such as their age, gender, race, ethnicity, or socioeconomic status [14,15]. The intersection of multiple marginalized identities or characteristics can compound stigma and its adverse health consequences [16-20].

Despite documented harms of stigma and its internalization, few interventions exist to combat internalized health-related stigma. Intervention approaches that have been proposed include peer support and psychological counseling [21]. Some studies have found benefits of these interventions when tested to reduce internalized stigma due to mental illness, HIV, or obesity [22-26]. However, such interventions have been relatively unstudied for most health conditions (eg, cancers, skin diseases) [6,27]. Health-related stigma is typically studied in disease-specific silos, despite many similarities in the presentation and impacts of stigma across health conditions [21]. Given the lack of interventions for internalized stigma, an intervention that reduces internalized stigma across different health conditions—rather than a disease-specific intervention that benefits only 1 patient population—could have the potential to enhance dissemination of and increase access to psychosocial support for adults with chronic health conditions who have internalized stigma. A “transdiagnostic” intervention could also address the needs of adults with multiple chronic health conditions, as well as leave space to address other intersecting marginalized identities.

Transdiagnostic approaches are commonly used in mental health care [28]. For example, skills groups can teach evidence-based coping strategies (eg, mindfulness, cognitive restructuring) to individuals with different types of mental health concerns within the same group [29,30]. A transdiagnostic framework is less commonly applied to the treatment of chronic physical health conditions, due in part to the importance of understanding the specific etiology and pathophysiology of each health condition and the unique needs of each patient population. However, developing a psychological intervention that addresses stigma transdiagnostically, across different stigmatized health conditions, could have several benefits, including saving time and resources. Disease-specific interventions also require individual clinics or specialists to deliver treatment, while a transdiagnostic intervention could be delivered in a centralized manner (eg, through referrals from different specialties within a health system) and by a mental health provider who does not need to have expertise in any 1 particular health condition. Including individuals with different stigmatized health conditions within the same groups could also serve to further destigmatize their conditions by highlighting commonalities and shared experiences among them, thus emphasizing the universal nature of stigma and leading individuals with chronic health conditions to feel less isolated or “different” from others [8,31]. However, possible downsides of this approach include reduced capacity to tailor interventions to address the specific challenges faced by patients with certain health conditions, and the potential for individuals who have internalized stigma to feel uncomfortable discussing their health conditions with individuals who do not share their same diagnoses. Given the novelty of this proposed transdiagnostic approach to addressing health-related stigma, preliminary research to assess feasibility and acceptability is warranted.

The present study was an early-phase (stage 1 [32]) investigation of a transdiagnostic, online, group-based psychological intervention designed to reduce internalized health-related stigma across different stigmatized chronic physical health conditions. The primary aim of this study was to determine the feasibility and acceptability of the intervention. The study was conducted in preparation for a larger planned randomized controlled clinical trial that will test the efficacy of the intervention in comparison to general peer support and a waitlist control.

## Methods

### Participants and Procedures

Participants were eligible if they had one or more of the following health conditions: obesity; type 1 or type 2 diabetes; skin disease (eg, psoriasis, eczema, or vitiligo); HIV; chronic pain; or cancer (including in remission). These conditions represent a range of dimensions important to stigma (eg, perceived controllability, contagion, and concealability). Inclusion criteria also included self-reported perceptions and internalization of health-related stigma. The latter criterion was determined by a cutoff score of 3.4 or higher on the Internalized Health-Related Stigma (I-HEARTS) Scale [33] (described below), with confirmation by clinical interview that participants’ health condition(s) negatively affected how they thought and felt about themselves. These criteria were included to ensure that the stigma intervention would be relevant to participants and that the study would include individuals who could benefit most from it. Study candidates were excluded if they currently or in the past 3 months received psychotherapy or psychosocial or peer support or if they had been hospitalized for psychiatric reasons in the past 6 months, due to potential confounding effects of treatment. Additional exclusion criteria were current, active suicidal thoughts or a reported suicide attempt within the past year; a current alcohol or substance use disorder that required immediate treatment; severe progression of disease (eg, end-of-life) or undergoing current acute, intensive treatment (eg, chemotherapy or radiation); or a current or past thought disorder, psychosis, or unmanaged bipolar disorder, as these conditions require a higher level of care and could interfere with the ability to participate in group meetings.

Participants were recruited primarily from the University of Florida (UF) Health system. Using a database of patients who agreed to be contacted for research studies, emails describing the study were sent to adults with eligible health conditions (in batches with equal numbers across the 6 health condition categories) with a link to a pre-screening questionnaire to determine initial eligibility. Additional efforts were made to recruit participants from community health organizations and through referrals from UF Health clinics. Study candidates who were initially eligible were contacted to conduct a phone screening interview with a research coordinator, followed by a virtual interview with the principal investigator to confirm eligibility and provide informed consent. During the virtual interview, the principal investigator discussed with participants their comfort sharing information about stigma in a group setting with others who may have different health conditions from them, as well as issues of privacy, confidentiality, and other concerns that may arise from participating in group treatment. Privacy, confidentiality, and group guidelines about treating others with respect were also reviewed during the first group meeting, with the goal of creating a safe and supportive environment.

In the week before starting the intervention, participants completed baseline questionnaires online. Participants received 12 weekly, 50-minute virtual treatment sessions led by the principal investigator (a licensed psychologist) and co-led by a doctoral student in clinical psychology. Participants who were absent from group sessions were offered brief make-up phone sessions with the group leader or co-leader within 1 week of the missed session. Participants completed questionnaires again during week 12. Study staff tracked adverse events reported by participants (none were determined to be related to the study).

### Ethical Considerations

Study procedures were approved by the University of Florida Institutional Review Board (IRB202201862). Informed consent was obtained from all participants. Participants were assigned identification numbers so that study data could be analyzed in deidentified form, with only select study staff having access to identifying information. Participants were compensated with a US $50 electronic payment or debit card after completing week 12 questionnaires.

### Intervention

The intervention was adapted from the Weight Bias Internalization and Stigma (Weight BIAS) Program, which was developed and tested for the purpose of reducing internalized weight stigma among adults with obesity [34]. The Weight BIAS program is a group intervention (incorporating peer support) that uses evidence-based principles and strategies from cognitive behavioral therapy and third-wave behavioral therapies, tailored specifically to address internalized stigma. To adapt the intervention, session topics and skills were largely retained, but session content was made generalizable to other health conditions beyond obesity by including examples relevant to other health conditions (eg, examples of negative stereotypes or self-critical thoughts that individuals with different health conditions may report). New topics and skills were also added to address facets of stigma that may not have been relevant to adults with obesity (eg, the topic of disclosure for individuals with concealable health conditions). All sessions were designed to be transdiagnostic—rather than having health condition-specific sessions or modules—so that individuals with any of the included health conditions could potentially benefit from each session. Some sessions also included prompts to discuss interactions between stigma due to health conditions and other identities.

Two Advisory Boards were formed to participate in the intervention adaptation: a Community Advisory Board comprised of adults representing each of the 6 categories of health conditions included in the study, and a Health Professional Advisory Board of researchers and clinicians who specialized in these health conditions. Advisory Board meetings were held online, in small groups, to discuss ideas for the intervention and provide feedback on a preliminary outline of session topics. Advisory Board members also provided feedback on the study design and structure (eg, including individuals with different stigmatized health conditions within the same treatment groups) in order to prevent the inadvertent perpetuation or exacerbation of stigma within the intervention or research study. Formal feedback on session topics and materials was obtained via a Qualtrics survey to determine acceptability and relevance of the content to each patient population. Changes were made based on this feedback (including incorporating specific examples of stigma provided by Advisory Board members to include in the treatment manual), and final treatment materials (including the treatment manual and electronic participant handouts) were disseminated to Advisory Board members prior to starting the study.

### Measures and Analyses

Feasibility was assessed for recruitment, retention, and adherence. The recruitment goal was 10 participants, with representation across the 6 categories of health conditions. This criterion was based on recommendations for optimal psychotherapy group sizes [31,35], and because the larger planned clinical trial will aim to recruit individuals for groups of 8‐10 participants across these 6 types of health conditions. Other metrics of feasibility included achieving participant retention of at least 80% and an average session attendance rate of at least 70% (calculated by averaging the total number of sessions attended by participants, both inclusive and exclusive of makeup sessions) [36,37].

Treatment acceptability was assessed with a questionnaire completed by participants at week 12 [25]. In total, 4 items assessed from 1 (not at all) to 7 (extremely) how helpful and acceptable the program was, how much participants liked the program, and how satisfied they were with the program; these items were averaged to produce an overall acceptability score (Cronbach α=.93). Participants also rated on the same scale how likely they would be to recommend the program to others. In addition, they rated from 1 (not at all) to 7 (very much so) the extent to which they learned new things, changed their attitudes about themselves, and the program helped them manage their health conditions. Participants rated on the same scale how much they learned 10 specific skills in the program (eg, challenge myths and stereotypes about health conditions; see Table S1 in Multimedia Appendix 1 for a full list of skills; Cronbach α=.94), and rated from 1 (never) to 5 (frequently) how often they used these skills from the program during the prior 12 weeks (Cronbach α=.84). Participants were also asked a few open-ended questions to give brief, additional feedback about their favorite part of the program, what they did not find helpful, and what they believed they were taking away from the program. Given the brevity of responses elicited and limited qualitative data, a formal content analysis was not planned or conducted, but responses were reviewed and representative quotes were extracted.

As part of feasibility testing, participants completed a battery of pre- and post-treatment questionnaires, as they would in a larger efficacy trial. This battery included the I-HEARTS Scale to assess internalized health-related stigma, which was previously adapted from the Internalized Stigma of Mental Illness Scale [38] and validated in a large sample of adults with varying chronic health conditions, showing strong psychometric properties [33]. The cutoff score of 3.4 out of 7 was established in prior research to identify clinically significant levels of internalized stigma [33] and was used to determine eligibility in the present study. The scale produces a total score, as well as 3 subscale scores for Perceived and Anticipated Stigma, Stereotype Application and Self-Devaluation, and Stigma Resistance.

Other measures completed by participants included the Internalized Shame Scale (with subscales for shame and self-esteem [39]) and the UCLA Loneliness Scale (version 3 [40]), given that these constructs are highly relevant to internalized stigma. Mental health was assessed with the Patient Health Questionnaire-9 (to assess depression [41]); the Generalized Anxiety Disorder-7 [42]; and the 10-item Severity Measure for Social Anxiety Disorder [43]. The 4-item Perceived Stress Scale assessed general stress [44]; several subscales of the Revised Illness Perceptions Questionnaire assessed disease-related quality of life [45]; and the 12-item Short Form Health Survey (SF-12 [46]) and the CDC Healthy Days Core Measure [47] assessed general mental and physical health-related quality of life. These outcome measures will be relevant to include in a larger efficacy trial due to links between internalized stigma and poor mental and physical health outcomes. Although primarily included for feasibility testing, exploratory completer’s analyses examined effect sizes from paired t-tests conducted between baseline and week 12.

For descriptive purposes, participants completed a questionnaire about their health conditions (eg, their diagnoses, visibility of the health conditions, and severity of symptoms) and were asked to report their demographic characteristics. They also completed the Everyday Discrimination Scale [48] to identify reasons for perceived discrimination.

## Results

Figure S1 in Multimedia Appendix 1 displays the participant flow chart. One study candidate recruited from a community group completed a pre-screening survey in November 2023. Email invitations to patients in the UF Health database were first sent in early December 2023, and the first participant was consented in early January 2024, with recruitment ending in mid-January. Altogether, the goal of enrolling 10 participants was met within approximately 6 weeks. Two of these participants did not start the intervention due to a personal or family medical event, respectively.

Table S2 in Multimedia Appendix 1 presents characteristics of the 10 enrolled participants, and Table 1 presents characteristics of the 8 participants who received the intervention. Participant characteristics did not differ between those who did versus did not receive the intervention. Participants predominantly identified as non-Hispanic, White, female, heterosexual, married, and either retired or receiving disability benefits, with a mean age of 60 years. Participants reported some college education on average, and household income ranged from <US $10,000 to US $50,000‐74,999. The most common health conditions reported were chronic pain and obesity, and most participants reported having 3 or more of the 6 different types of health conditions. Most participants indicated that their health conditions were visible and were moderate in severity overall. Apart from the 6 main categories of health conditions, other health conditions reported by participants included Hashimoto’s disease, liver disease, heart disease, hypertension, autoimmune hepatitis, dystonia, dysphonia, and migraines.

**Table 1.** Characteristics of adults with stigmatized chronic health conditions who received the 12-week internalized health-related stigma intervention (*N*=8).

Variables	Participants (N=8), n (%)
Age (years), mean (SD); range	59.1 (8.9); 47‐69
Sex, n (%)	
Female	6 (75.0)
Male	2 (25.0)
Race and ethnicity, n (%)	
Non-Hispanic White	6 (75.0)
Non-Hispanic Black	2 (25.0)
Sexual orientation, n (%)	
Heterosexual or straight	7 (87.5)
Asexual	1 (12.5)
Marital status, n (%)	
Single (never married)	2 (25.0)
Married	5 (62.6)
Divorced	1 (12.5)
Employment status, n (%)	
Retired	3 (37.5)
On disability	3 (37.5)
Employed part-time	1 (12.5)
Unemployed	1 (12.5)
Annual household income (US) $, n (%)	
Less than $10,000	1 (12.5)
$10,000-$24,999	1 (12.5)
$25,000-$34,999	1 (12.5)
$35,000-$49,999	3 (37.5)
$50,000-$74,999	1 (12.5)
Prefer not to answer	1 (12.5)
Education (years), mean (SD)	14.1 (2.5)
Health conditions, n (%)	
Obesity	5 (62.5)
Diabetes	
Type 1	0 (0)
Type 2	3 (37.5)
Skin disease	2 (20.0)
HIV	1 (10.0)
Chronic pain	7 (87.5)
Cancer (in remission)	2 (20.0)
One of the above health conditions	2 (20.0)
Two of the above health conditions	1 (10.0)
Three or more of the above health conditions	5 (62.5)
Participants who reported additional health conditions not listed above	6 (75.0)

All 8 of the participants who attended at least 1 session of the intervention completed the week 12 questionnaires in April 2024, thus meeting the overall retention goal of 80%. Among these 8 participants, the average attendance rate was 95.8% (11.5 sessions) when including makeup sessions, or 83.3% (10 sessions) when excluding makeup sessions—all exceeding the goal of an average 70% attendance rate.

**Figure 1.**
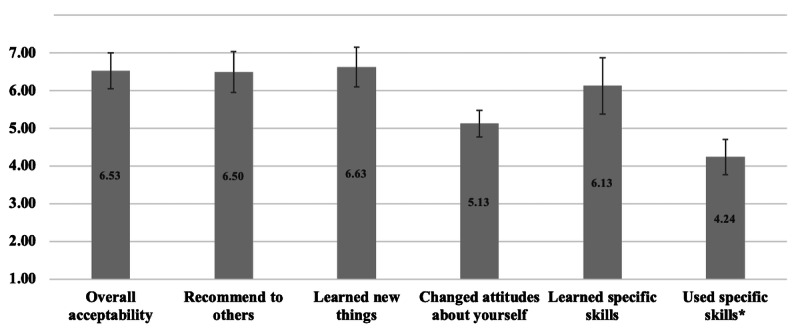
Treatment acceptability ratings of the internalized health-related stigma intervention. *Ratings for “Used specific skills” were on a scale from 1 (never) to 5 (frequently). All other items were rated on a 1‐7 scale.

Treatment acceptability ratings were high (Figure 1), with an average overall acceptability rating of 6.5 (SD 0.5) on a 1‐7 scale. Open-ended responses were unilaterally positive and cited the following program benefits: connecting with others who had similar experiences, feeling less alone, learning coping strategies, and being less judgmental of themselves. Representative quotes are included in Table S3 in Multimedia Appendix 1.

Table 2 summarizes changes in continuous measures. Effect sizes were medium to large for reductions in internalized stigma as measured by the I-HEARTS Scale and its subscales. Effect sizes were also medium to large for improvements in loneliness, stress, and some aspects of disease-related and general mental health-related quality of life. Effect sizes were smaller for other mental health changes and minimal for changes in physical health-related quality of life. Participants reported many reasons for discrimination, with their health conditions reported most frequently (Table 3).

**Table 2.** Baseline and week 12 scores on continuous measures assessed before and after the internalized health-related stigma intervention. Higher scores indicate worse outcomes on all measures, except for the Self-Esteem Subscale of the Internalized Shame Scale, the Personal Control Subscale of the Illness Perceptions Questionnaire-Revised, and the SF-12.

Measure	Scale range	Baseline scores, mean (SD)	Week 12 scores, mean (SD)	Change in scores, mean (SD), 95% CI	Cohen *d*
I-HEARTS^a^ total score	1‐7	4.49 (0.71)	3.84 (1.22)	−0.65 (1.45), −1.86 to 0.56	−0.65
Perceived and Anticipated Stigma Subscale	1‐7	4.54 (0.74)	3.82 (1.33)	−0.72 (1.64), −2.09 to 0.65	−0.67
Stereotype Application and Self-Devaluation Subscale	1‐7	4.53 (1.63)	3.31 (1.82)	−1.22 (1.96), −2.86 to 0.42	−0.71
Stigma Resistance Subscale (reverse-scored)	1‐7	4.22 (1.29)	3.19 (1.25)	−1.03 (1.69), −2.44 to 0.38	−0.81
Internalized Shame Scale					
Shame	0‐96	51.25 (18.01)	43.88 (18.23)	−7.38 (13.68), −18.81 to 4.06	−0.41
Self-esteem	0‐24	12.88 (3.44)	13.50 (4.04)	0.63 (5.10), −3.64 to 4.89	0.17
UCLA^b^ Loneliness Scale	20‐80	53.75 (9.77)	46.25 (10.90)	−7.50 (7.35), −13.64 to −1.36	−0.73
Patient Health Questionnaire-9	0‐27	11.63 (6.30)	10.25 (5.95)	−1.38 (3.02), −3.90 to 1.15	−0.23
Generalized Anxiety Disorder-7	0‐21	9.25 (6.78)	7.00 (4.66)	−2.25 (3.99), −5.59 to 1.09	−0.39
Social Anxiety Disorder Questionnaire	0‐40	11.50 (9.09)	9.75 (7.38)	−1.75 (7.31), −7.86 to 4.36	−0.21
Perceived Stress Scale	0‐16	9.00 (2.56)	6.63 (2.77)	−2.38 (2.13), −4.16 to −0.59	−0.89
Illness Perceptions Questionnaire-Revised					
Consequences	1‐5	4.33 (0.49)	4.00 (0.37)	−0.33 (0.35), −0.62 to −0.05	−0.77
Personal control	1‐5	3.56 (0.98)	3.75 (0.46)	0.19 (1.30), −0.90 to 1.27	0.24
Illness coherence	1-5	3.55 (0.64)	3.43 (0.55)	0.13 (0.94) −0.91 to 0.66	−0.21
Emotional representations	1‐5	3.73 (0.86)	3.46 (0.61)	−0.27 (0.66), −0.82 to 0.28	−0.36
SF-12^c^					
Mental health	0‐100	37.63 (8.47)	43.02 (7.17)	5.40 (10.35), −3.25 to 14.05	0.69
Physical health	0‐100	29.02 (9.20)	28.96 (10.82)	−0.06 (5.55), −4.70 to 4.58	−0.01
CDC^d^ Unhealthy days index	0‐30	26.50 (6.82)	25.00 (9.32)	−1.50 (7.76), −7.99 to 4.99	−0.18

a I-HEARTS: Internalized Health-Related Stigma.

b UCLA: University of California, Los Angeles.

c SF-12: 12-item Short Form Health Survey.

d CDC: Centers for Disease Control and Prevention.

**Table 3.** Reasons for discrimination identified by adults with stigmatized chronic health conditions with the Everyday Discrimination Scale (*N*=8). Frequencies reflect participants who endorsed these reasons in response to any item on the Everyday Discrimination Scale.

Reason for discrimination	Participants, n (%)
Your ancestry or national origins	1 (12.5)
Your race	2 (25)
Your gender	2 (25)
Your age	4 (50)
Your height	1 (12.5)
Your weight	5 (62.5)
Some other aspect of your physical appearance	4 (50)
Your education or income	2 (25)
Your health condition	7 (87.5)
Your physical disability	5 (62.5)
Your shade of skin color	1 (12.5)
Other	2 (25)

## Discussion

This early-phase intervention study provides preliminary support for the feasibility and acceptability of a new group-based psychological intervention designed to reduce internalized health-related stigma among adults with different stigmatized chronic health conditions. Results showed successful recruitment, in a short period of time, of adults with obesity, diabetes, skin diseases, HIV, chronic pain, and cancers to participate in a 12-week online intervention study. Retention and session attendance rates and ratings of treatment acceptability were high. Altogether, study results support the potential to recruit participants in a similar manner for a larger trial.

The successful recruitment, retention, and treatment adherence suggest that individuals with chronic health conditions were open to engaging in group discussions of stigma with others who had different health conditions from their own. This was also reflected in open-ended treatment acceptability questionnaire responses and in Advisory Board meeting discussions, especially among Board members living with different kinds of health conditions. A prominent feature of internalized stigma is feeling “othered,” isolated from society, and alone [8]. Group treatment with individuals who vary in their health conditions may serve to highlight to individuals who have internalized stigma that they are not alone [31] and to facilitate finding commonalities with others who do not share their stigmatized health conditions. Notably, individuals who may not have felt comfortable participating in a group of individuals with differing health conditions likely would not have responded to recruitment advertisements, and thus may not have been represented in the sample. It is possible that individuals with the highest levels of internalized stigma may be most uncomfortable with this treatment format. Nevertheless, this preliminary study suggests that, for many individuals with chronic health conditions who have internalized health-related stigma, the treatment paradigm pilot-tested in this study was considered acceptable.

Measures of internalized stigma and relevant mental health and quality of life constructs were primarily included in the study for the purpose of feasibility testing. Pre-post treatment change scores indicated medium to large effects for many variables, including internalized stigma. This study was not designed to test the efficacy of the intervention, and these effect sizes should be interpreted with caution given the very small sample size and limited generalizability. Prior studies testing interventions for internalized health-related stigma have shown a wide range of effect sizes, some of which have been challenging to interpret due to small sample sizes, lack of control groups, and other design limitations [6,21,23,49-51]. Still, several promising interventions have been rigorously tested in recent years for specific health conditions [22-26,52], although such interventions are lacking for most physical health conditions [1,21,51,53,54]. Further testing of this transdiagnostic intervention in an adequately powered randomized controlled trial is needed in order to be able to compare its effect sizes to those of disease-specific stigma interventions.

Almost all participants reported experiencing discrimination due to their health conditions, which was not surprising given that participants were selected for having high levels of internalized stigma. However, participants reported many other reasons for perceived discrimination as well, including factors related to health (disability, weight) and other aspects of their identities (eg, age, race, and gender). These findings highlight the potential utility of applying an intersectional lens to interventions for health-related stigma, in order to acknowledge and address how interactions among different forms of stigma may affect adults with chronic health conditions [15].

The generalizability of the present work is limited in many respects. The study was conducted at a single site, so geographic location was limited, as was the age, gender, racial, and ethnic diversity of the small sample. No control group or randomization was used in the present research. Future testing in a larger efficacy trial is needed to determine the potential benefits of this transdiagnostic intervention for internalized stigma, mental health, and quality of life. This study also focused on stigmatized physical health conditions only; the stigma of mental health conditions was not an intended target for the intervention, in part due to the extensive research that has been conducted on addressing mental health stigma [55], in addition to the exclusion criterion of individuals currently or recently receiving psychotherapy (due to its potentially confounding effects with the treatment provided). Further research is needed to determine if one transdiagnostic stigma intervention could be applied to address both mental and physical health stigma, or if tailoring would be needed to address mental versus physical health concerns. More work is also needed to understand how to best balance the importance of meeting the unique needs of each patient population, while also leveraging knowledge and commonalities across health conditions to enhance dissemination of support to individuals who have internalized health-related stigma. Finally, in addition to interventions for intrapersonal-level interventions such as this, structural- and interpersonal-level interventions are needed to reduce public stigma and prevent the internalization of stigma and its negative associated health outcomes [56].

In conclusion, this preliminary study of a transdiagnostic, online, group-based psychological intervention for internalized health-related stigma showed strong evidence of feasibility and acceptability. This represents a first step in investigating this intervention, which will be followed by a randomized controlled trial to test its effects on internalized stigma. With further testing, this work could have the potential to shift the paradigm for how stigma is addressed and for how psychosocial support interventions may be more broadly disseminated to adults with chronic health conditions.

## Supplementary material

10.2196/69548 Multimedia Appendix 1 Additional information about: skills rated in treatment acceptability questionnaire (Supplemental Table S1); characteristics of all 10 enrolled participants (Table S2); representative quotes from open-ended responses in treatment acceptability questionnaire (Table S3); and flow chart of participants enrolled in the study (Figure S1).

## Abbreviations

I-HEARTS: Internalized Health-Related Stigma
Weight BIAS: Weight Bias Internalization and Stigma
