# Supplementary material for: Feasibility and Acceptability of a Psychological Intervention for Internalized Health-Related Stigma Among Adults With Chronic Health Conditions: Preliminary Investigation
Source: JMIR Form Res. 2025 Jul 29;9:e69548. doi: 10.2196/69548 (PMC12308099; doi:10.2196/69548)

Supplemental Table S1. Skills rated for learning and use in treatment acceptability questionnaires completed by adults with stigmatized chronic health conditions who completed the 12-week internalized health-related stigma intervention

| Challenge myths and stereotypes about my health condition(s) |
| --- |
|  |
| Identify the relationships between my thoughts, feelings, and health management behaviors |
|  |
| Reduce negative thoughts and critical self-talk about my health condition(s) |
|  |
| Reframe negative situations and thoughts related to my health condition(s) in a more positive or accurate way |
|  |
| Ask others to change their behavior related to my health condition(s) (for example, to stop making negative comments about my health |
|  |
| Feel empowered to stand up for myself if someone treats me poorly because of my health condition(s) |
|  |
| Increase confidence in my ability to achieve or stick with my life goals |
|  |
| Increase confidence in my ability to achieve or stick with my health behavior goals |
|  |
| Increase self-compassion concerning my health condition(s) |
|  |
| Increase self and/or body acceptance |
|  |
| Talk with others about negative societal attitudes toward and treatment of people with my health condition(s)* |

Note. *This skill was rated for use only.

Supplemental Table S2. Characteristics of adults with stigmatized chronic health conditions who enrolled in the study to receive a 12-week intervention targeting internalized health-related stigma (*N*=10)

| **Variable** | **Mean ± Standard Deviation or N (%)** |
| --- | --- |
| Age (years) | 60.0 ± 8.1 (range: 47-69) |
| Gender |  |
| Female | 8 (80%) |
| Male | 2 (20%) |
| Race /Ethnicity |  |
| Non-Hispanic White | 8 (80%) |
| Non-Hispanic Black | 2 (20%) |
| Sexual Orientation |  |
| Heterosexual or Straight | 9 (90%) |
| Asexual | 1 (10%) |
| Marital Status |  |
| Single (Never Married) | 2 (20%) |
| Married | 6 (60%) |
| Divorced | 1 (10%) |
| Widowed | 1 (10%) |
| Employment Status |  |
| Retired | 4 (40%) |
| On Disability | 3 (30%) |
| Employed Full-Time | 1 (10%) |
| Employed Part-Time | 1 (10%) |
| Unemployed | 1 (10%) |
| Annual Household Income |  |
| Less than $10,000 | 1 (10%) |
| $10,000-$24,999 | 1 (10%) |
| $25,000-$34,999 | 1 (10%) |
| $35,000-$49,999 | 4 (40%) |
| $50,000-$74,999 | 2 (20%) |
| Prefer not to answer | 1 (10%) |
| Education (years) | 14.2±2.4 |
| Health Conditions |  |
| Obesity | 7 (70%) |
| Diabetes |  |
| Type 1 | 0 |
| Type 2 | 5 (50%) |
| Skin Disease | 2 (20%) |
| HIV | 1 (10%) |
| Chronic pain | 8 (80%) |
| Cancer (in remission) | 2 (20%) |
| One of the above health conditions | 2 (20%) |
| Two of the above health conditions | 2 (20%) |
| Three or more of the above health conditions | 6 (60%) |
| Participants who reported additional health  conditions not listed above | 7 (70%) |

Supplemental Table S3. Representative quotes from open-ended responses in treatment acceptability questionnaire, completed by adults with stigmatized chronic health conditions following an intervention targeting internalized health-related stigma. Questions asked about favorite parts of the program and what participants were taking away from the program.

| Program Benefits Identified by Participants | Representative Quotes |
| --- | --- |
| Connecting with others who had similar experiences | “Hearing the ongoing stories/sagas of the other participants… There is a kind of bonding or companionship that seems to have developed… In this group it has been enlightening to hear and share so that I have become a little more aware of what some of what those really difficult ‘something’ medical issues are and the challenges resulting from that.”  “connecting with other people experiencing the same problems that I have”  “knowing that there’s others who have had similar issues” |
| Feeling less alone | “I am not alone”  “seeing that I’m not alone in this journey has been wonderful” |
| Learning coping strategies | “The handouts will continue to be a helpful resource moving forward. I will be referring to them to remind myself of what we learned. They are concise and clear. In general, variations of rethinking, re-framing, rewording, etc have been helpful. Coping strategies like breathing exercises are effective and can be used anytime, anywhere. Having the cognitive distortions all laid out and defined is helpful. The interconnections between Thoughts, Feelings and Behaviors is helpful. The DEAR and SMART acronyms are helpful. Skills that can be used with not only medical providers but also anyone we interact with will be useful.”  “I am taking away coping mechanisms”  “learning new ways to deal with my issues”  “more beneficial ways to cope with others” |
| Being less judgmental of themselves | “I think I’ll move forward being less judgmental of myself, using non-judgmental self-talk”  “more respect for myself. not to be so hard on myself” |

Supplemental Figure S1. Flow chart of participants with stigmatized chronic health conditions for a study testing an online, transdiagnostic intervention designed to reduce internalized health-related stigma


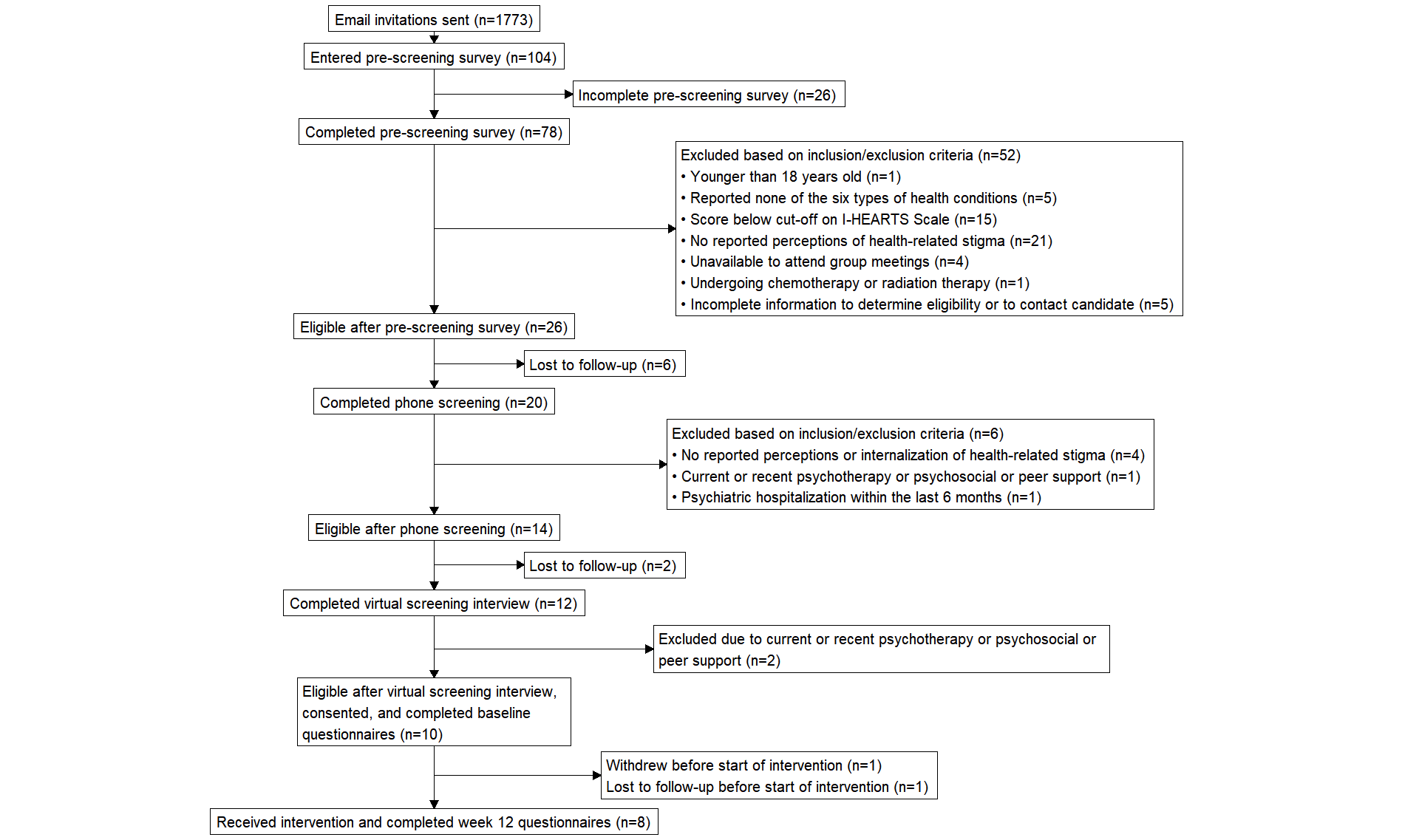

Supplement: Multimedia Appendix 1 [file formative-v9-e69548-s001.doc]
